# Implications for Welfare, Productivity and Sustainability of the Variation in Reported Levels of Mortality for Laying Hen Flocks Kept in Different Housing Systems: A Meta-Analysis of Ten Studies

**DOI:** 10.1371/journal.pone.0146394

**Published:** 2016-01-06

**Authors:** Claire A. Weeks, Sarah L. Lambton, Adrian G. Williams

**Affiliations:** 1 School of Veterinary Sciences, University of Bristol, Langford, Bristol, United Kingdom; 2 School of Energy, Environment and Agri-food, Cranfield University, Bedford, United Kingdom; Harvard University Faculty of Arts and Sciences, UNITED STATES

## Abstract

Data from ten sources comprising 3,851 flocks were modelled to identify variation in levels of mortality in laying hens. The predicted increase with age was curvilinear with significant variation between the seven breed categories. Mortality was higher in loose housing systems than in cages and variable within system, confirming previous reports. Cumulative mortality (CM) was higher in flocks with intact beaks (χ2 = 6.03; df 1; p = 0.014) than in those with trimmed beaks. Most data were available for free-range systems (2,823 flocks), where producer recorded CM at 60–80 weeks of age averaged 10% but with a range from 0% to 69.3%. Life cycle assessment showed that the main effect of increased levels of hen mortality is to increase the relative contribution of breeding overheads, so increasing environmental burdens per unit of production. Reducing CM to levels currently achieved by the 1st quartile could reduce flock greenhouse gas emissions by as much as 25%. Concurrently this would enhance hen welfare and better meet the expectation of egg consumers. More research to understand the genetic x environment interaction and detailed records of the causes of mortality is required so that improved genotypes can be developed for different systems and different breeds can be better managed within systems.

## Introduction

Conventional cages have been outlawed in Europe since 2012 (EU Directive 1999/74/EC). Although globally the prevalent system, the move away from keeping hens in conventional battery cages is extending beyond Europe to countries such as Australia, New Zealand, Canada and the USA. This follows increasing recognition of the extreme spatial and behavioural restriction these cages impose on hens, possibly offsetting their advantages in terms of hygiene and reduced exposure to potential disease causing organisms [1, 2, 3]. Therefore, increasing numbers of laying hens are being kept in non-cage housing systems in large groups of several thousand birds. Contrary to consumer expectation, however, surveys have frequently found the mean levels of mortality to be both high and greater in these systems than in cage systems. For example, one study found 6.9% mortality in 25 free-range flocks [4], whereas another [5] reported a mean of 4.2 x and 2.0 x greater levels of mortality [birds found dead by 66–72 weeks of age] in seven barn systems and seven free range systems respectively, compared with six furnished cage systems. In New Zealand flocks, mean mortality rates to 63 weeks of 2.9% in furnished cages, and 6.3% in free range systems were recorded [6]. In a larger survey [7] of UK data from 1,486 flocks in 2009 we found that mortality rates by the end of lay in free-range flocks (mean: 9.5%) were significantly greater than in flocks kept in cages (both furnished and conventional; mean: 5.4%). These figures were based on producer records on the Food Chain Information form completed a median of 7 days before slaughter, thus slightly underestimating the final cumulative mortality on farm.

The furnished cage system was developed to enable the needs of laying hens for nesting, perching and to some extent dustbathing behaviour [8] to be met whilst also retaining good hygiene and small group sizes (e.g. in Sweden typically 8–10 birds [9]). As yet there is limited published information evaluating the welfare of hens in larger colony sizes of 80 birds or more per cage; moreover to several welfare organisations ‘a cage is a cage’ and pressure is mounting to extend current bans on conventional cages to include furnished cages as well [10].

If increasing numbers of laying hens are to be kept in non-cage systems that frequently, if not inherently, have higher levels of associated mortality then there are negative implications not only for bird health and welfare but also for food security and sustainability resulting from the reduced productive output of such flocks. These include fewer eggs produced per unit of land and higher burdens on the environment per unit production. For example, higher greenhouse gas and ammonia emissions, and cumulative energy use from free-range than caged systems were found in an analysis that used average industry mortality rate data and so could not readily address the impacts of different mortality rates [11].

This paper therefore examines the impact of on-farm cumulative mortality (CM) using data from European producer records for commercial flocks kept in different housing systems. The analysis uses raw data from ten published and unpublished studies (approximately 45 million laying hens). This study aims to improve the accuracy and reliability of estimates of levels of mortality and to identify some associated risk factors. Using the variation between flocks, it also predicts the consequences for food security and land use of depressed production associated with higher levels of mortality.

## Materials and Methods

### 2.1. Mortality data

Cumulative mortality data were collated from nine different UK sources, and one source that provided information from the Netherlands and Sweden (Table 1). Data sources were selected for relevance and in particular the availability of raw data. Data were provided from previously published scientific studies and unpublished sources such as farm assurance schemes.

**Table 1:** Summary of the studies from which cumulative mortality data were obtained and used for this analysis.

Study	Source	Reference	Details	No. of observations	Est. no. of flocks	Est. no. of farms
1	FAI Farms, Oxford, UK	Unpublished data	UK farms supplying McDonalds (2008–12)	970	970	342
2	University of Bristol	Unpublished data from study by [12]	Data from 11 FC and 12 FR flocks studied during transport to slaughter (2009–11)	23	23	16
3	Welfare Quality®	Unpublished data	The Netherlands and Sweden (2006–11)	119	119	119
4	University of Bristol	Sherwin et al [5]	A comparison of four different housing systems for laying hens	26	26	15
5	University of Bristol	Unpublished UFAW Summer studentship	Study examining time course of rates of mortality in free-range flocks	70	70	18
6	University of Bristol	Lambton et al [13]	A study of the efficacy of management strategies designed to reduce injurious pecking in free range laying hens.	133	133	57
7	University of Bristol	Unpublished data from RSPCA study	A study of range use in free range laying hens	36	36	21
8	University of Bristol	Nicol et al [14]	Experimental evaluation of the effects of stocking density and flock size in aviary systems on hen behaviour	36	36	1
9	Food Standards Agency	Raw data used in [7]	UK national data from 5 slaughter plants during 2009	1,501	1,501	770
10	AssureWel®	unpublished	Data collected by assurance scheme assessors at different ages in the UK	937	650	650
Total				3,851	3,564	2,009

The CM data were collected from 3,851 flocks between 2005 and 2012, at a range of ages (mean: 65.3 w, range: 16–208 w) and flock sizes (mean: 11,742, range: 6–172,500). Data came from seven breed categories: Columbian Black Tail; Hyline Brown; ISA Warren; Lohmann Brown; Shaver Brown; Other (commercial breeds which were not represented in large enough numbers to be included separately); and Traditional breeds. Henceforth these breeds are represented by randomly assigned codes A-G (n = 160; 1,239; 856; 78; 312; 211; and 594, respectively), unrelated to the order in which they are listed above. Six housing systems provided data: two in which the birds were confined in small groups within either conventional (battery) cages or furnished (colony) cages with 447 and 51 flocks respectively, and four loose-housed systems where birds within the flock could move around freely. The loose-housed systems included aviary, barn, free range and free range aviary (n = 31; 168; 2998; 125, respectively). Aviary denotes a house where the birds have access to several levels, or tiers, with litter provided at ground level; feed, water, perches and nests are provided on one or more of the other levels. Barn (also called single-tier) is a house with no access to the outdoors where the birds have litter at ground level and other resources on a raised, slatted area. Free range housing systems include static houses similar to barn housing but with low-level hatches called popholes that give access to the range (a field which may contain trees and other shelter) during daylight hours, and they also include smaller houses which can be moved but which also give access to the range during daylight. A separate category of ‘free-range aviary’ was chosen for aviary housing with range access, as the popholes are less visible and accessible to hens in multi-tier systems. Organic flocks legally have to have access to free-range and in general are kept in mobile or barn housing but aviary is possible; their feed and pasture have to be managed organically. More information on housing systems can be found at www.laywel.eu.

Data also came from beak trimmed and intact beak flocks (n = 801 and 228, respectively), and organic and non-organic flocks (n = 349 and 2,477, respectively). Data were missing for some variables within studies, and not all studies provided information on all variables, therefore analyses of some variables came from a smaller number of data sets.

The data are hierarchical: one of the studies included multiple data points from the same flock, and eight studies included data from more than one flock on the same farm. Data were examined in considerable detail and, where possible (e.g. where farms and flocks were named and/or numbered), each flock and each farm was given a unique identifier. However, this information was not clearly labelled in all studies, thus the number of farms and flocks are in some cases an estimate (Table 1). The hierarchical structure may not be perfect, as it is likely that some of the farms were visited in more than one study, there being a finite number in the UK, however each study would have used a different flock, thus reducing the possibility of duplication.

Multilevel models were generated using Stata 12.0 (StataCorp LP, Texas, USA) to reflect the hierarchical structure of the dataset: multiple visits to flocks within farms within studies. The response variable, CM, was square root transformed. All models included the time of year at which the CM data were collected: date was sine and cosine (sin(2*pi*(x/365)) and cos(2*pi*(x/365)), where x is the number of days on a given date since 1^st^ Jan 2005 (the first year for which we have data); i.e. on 2^nd^ Jan 2005 x = 1, 25^th^ Jan 2005 x = 24 etc.) transformed to account for the cyclical nature of annual patterns [15]. Both the sine and cosine transformed variables were included in all models to allow more flexibility in the curves generated. The explanatory variables: age; flock size; house type; breed; beak trim and organic status were each entered individually into the model to produce bivariable models (controlled for time of year as described above). Variables significant in bivariable models were then entered together into a multivariable model; any non-significant variables (p>0.05) were removed and the model was re-run. Thus a final model was produced with only significant variables.

Two-way interaction effects were examined where there was sufficient data to model each interaction category (i.e. not all house type / breed combinations were represented in the data) and where the interaction was considered to be of biological interest.

Sensitivity analysis was carried out to examine the effect of removing outlying data points, thus the analyses were repeated separately including only flocks of ≥500 birds, only flocks ≤100 weeks of age, and only flocks with CM ≤40%.

### 2.2. Environmental impacts

The environmental impacts of mortalities were calculated using Life Cycle assessment (LCA). This is a systematic method for accounting for the resources used and emissions released to the environment in the production of goods [16]. It was applied using a development of the systems-LCA model [11], which was derived from previous work [17]. The LCA was limited to free range non-organic production and used information from breed B, for which there was the greatest number of flocks (n = 1,239) in the CM dataset.

The systems-LCA model quantifies all the inputs needed to produce the functional unit of 1 kg of potentially marketable eggs. Weight (rather than number) is used to allow for the different weights of eggs produced by breeds, systems and over a production cycle. The inputs include feed, water, direct energy (for heating, lighting, ventilation etc.) and pullets. Pullet production is quantified through three generations, so that the complete industry is represented. The principal output is potentially marketable eggs, which omits those not laid in proper nest boxes in loose-housed systems. In addition, end of lay hens can contribute to the meat supply chain. Manure is usually managed as a soil conditioner and the balance of fertiliser value and management effort is calculated in a separate manure module, based on [18].

Environmental burdens are quantified in terms of emissions and resource use. Emissions are calculated as point source or diffuse processes and individual emissions are commonly aggregated into potentials for causing harm using factors taken from the University of Leiden database [19]. The ones reported here are for greenhouse gas emissions (GHGE), quantified as global warming potential (GWP) as CO_2_ equivalents (CO_2_e), which uses the 2007 IPCC GWP factors [20], acidification potential as SO_2_ equivalents and eutrophication potential as phosphate equivalents. Pesticide use (from arable operations) is quantified as dose-ha.

Resources are quantified as cumulative energy demand, in which all energy sources are traced back to primary energy in the ground, e.g. oil, uranium, and all extraction, refining and delivery overheads are included. Disparate inputs, e.g. diesel and building materials are quantified on unified scale of abiotic resource use using the antimony (Sb) scale [19]. Abiotic resources are non-biological and often non-renewable. Land occupation includes the main term of land used for crop production, together with housing and ranging land for the birds themselves.

#### 2.2.1 Anticipated environmental impacts of mortalities

At least one pullet must be reared for each laying hen entering a commercial laying flock and this is typically supported by three generations of breeder flocks. Pullet production can be regarded as a necessary overhead of producing eggs. In a laying flock, dead hens are not normally replaced, hence the inputs of electricity (e.g. lighting and ventilation) will increase per bird as the laying cycle progresses. Dead hens must be removed and managed in accordance with animal by-products regulations. This could be by on-site incineration, centralised incineration or rendering. Egg production, feed consumption and manure production stop at the point of death, and productivity may have been reduced prior to death.

If a flock had zero mortality, a maximum number of eggs would be produced per pullet reared to the point of lay. With higher mortalities (as in normal commercial practice), a smaller number of eggs are produced per pullet reared. Hence, from the life-cycle perspective, the proportion of the total burdens per egg produced that are attributable to pullet production goes up with CM.

#### 2.2.2. LCA Model development

In [11], the systems-LCA model used average industry values of egg number, average egg weight and CM for the analysis. With the focus in the current paper on CM, it was important to establish the effects of the timing of mortalities, because egg weights and productivity change with time and thus affect resource use, e.g. feed and electricity, and emissions during the production cycle. Some development was therefore made, using the CM data in this paper, to the systems-LCA model of [11] to ensure that changes over time were accurately quantified. This involved using the breed management guide to obtain the expected feed intake, egg production, hen liveweight and cumulative mortality range during the production phase for breed B in free range systems to 72 weeks. The details available allowed the effects the timing and magnitude of mortality to be determined by changing parameter values in the model. The mortality rates predicted by the LCA model were consistent with the separate analysis of the CM data (2.1), but not identical. The LCA results thus represent typical performance of a common strain housed in a free-range system.

#### 2.2.3. Data sources

Details of the egg production and feed characteristics of the breed (B) predominant in the CM dataset were taken from the breeder’s website and disaggregated to derive daily feed and water needs and egg outputs such that mortality could be analysed independently. This industry data source was in addition to the industry-derived data in [11], e.g. the cumulative mortalities of pullets and breeder hens used here were 3.5% and 7% respectively. Using this expected performance data meant that the shape of the CM curve was maintained irrespective of the actual final CM value at 72 weeks. As noted above this may have led to minor differences between this and the final model analysing CM in 2.1.

Breed average egg production data were only available for up to 72 weeks so the LCA was not projected after this, even though some CM data were available for older flocks. Incineration data were taken from the *Ecoinvent 2* database [21].

#### 2.2.4. Allocation

The partition of resources between eggs and hen body weight was on the basis of retained body protein and egg protein as used by [22, 23].

#### 2.2.5. Sensitivity analysis

A sensitivity analysis was conducted to compare the effect of changing key variables on the environmental impacts. The effects of different levels of mortality during lay were also assessed along with other main variables associated with egg production: egg yield, feed consumption, on-farm energy use and mortality in pullet rearing. Each variable was changed independently both up and down by 10% in 5% increments to check for linearity. Some additional changes were included to demonstrate relative magnitudes. These were conducted with 10% mortality as the baseline and responses were in proportion to the baseline values of 10% (which was the mean CM of FR flocks at 72 w in the dataset analysed in this paper).

## Results

### 3.1. Mortality analysis

**Fig 1:**
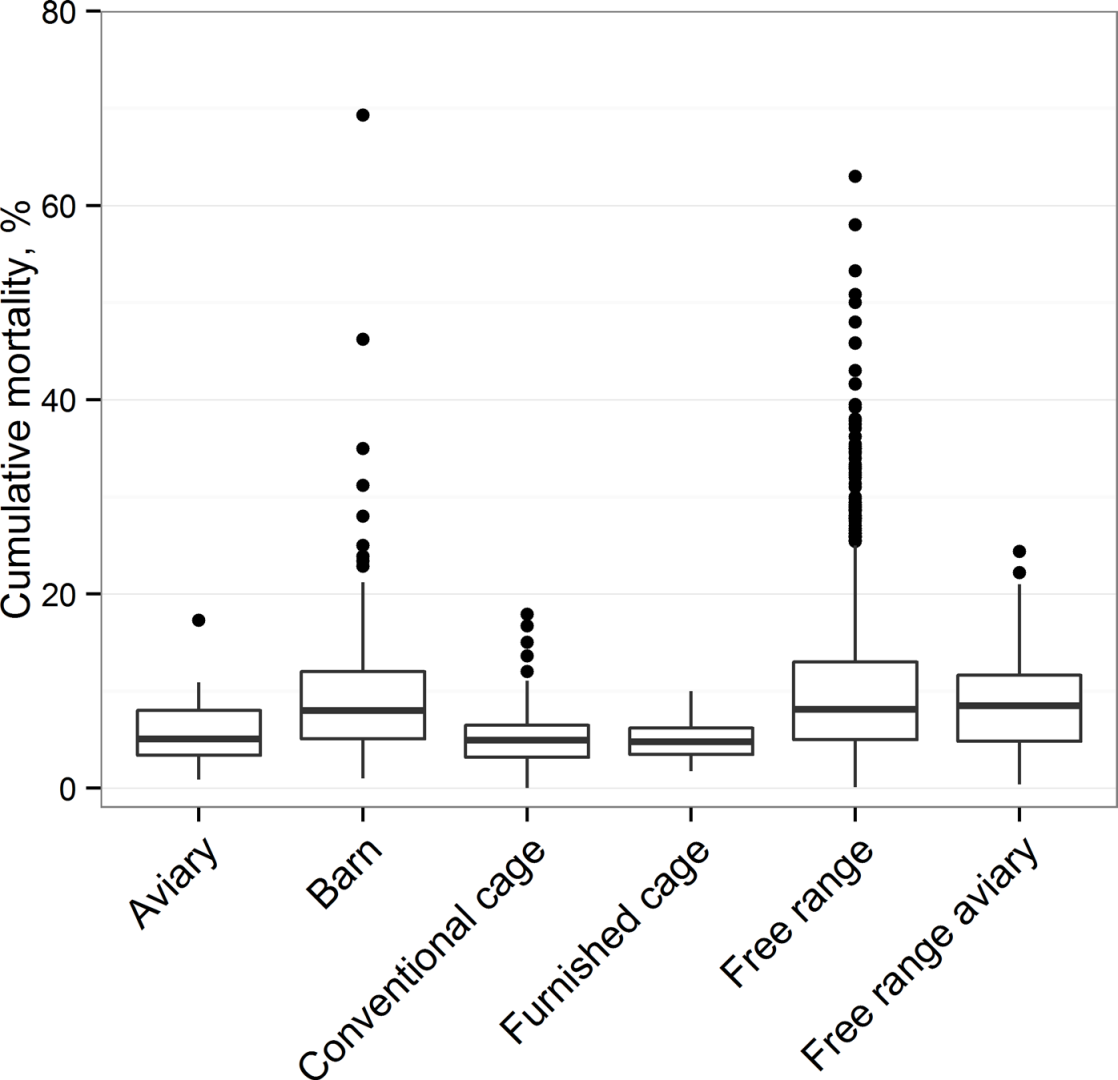
Box plots for mortality in each housing system between 60 and 80 weeks of age using the full data set from 10 studies (3,851 flocks).

The overall mean cumulative mortality (CM) was 7.89% (standard deviation 7.07) and ranged between 0 and 69.3% (Fig 1).

The bivariable associations between CM and each of the explanatory variables are shown in Table 2. All variables show highly significant associations with CM, which increased with age; decreased with flock size; was highest in flocks of intact beaked birds and organic flocks; and varied with both housing system and breed.

**Table 2:** Bivariable models of CM with each predictor variable.

Variable	n	χ^2^	df	p	Number of		
					Studies	Farms	Flocks
Time of year	3,534	22.7	2	<0.001	6	1,815	3,247
Age (quadratic)	3,758	468.1	2	<0.001	10	1,982	3,473
Flock size (quadratic)	3,765	27.3	1	<0.001	9	1,985	3,479
Housing system	3,820	87.8	5	<0.001	10	1,998	3,534
Breed	3,450	104.9	6	<0.001	9	1,840	3,163
Beak trim status	1,029	34.5	1	<0.001	4	660	781
Organic status	2,826	14.5	1	<0.001	9	1,638	2,450

**Fig 2:**
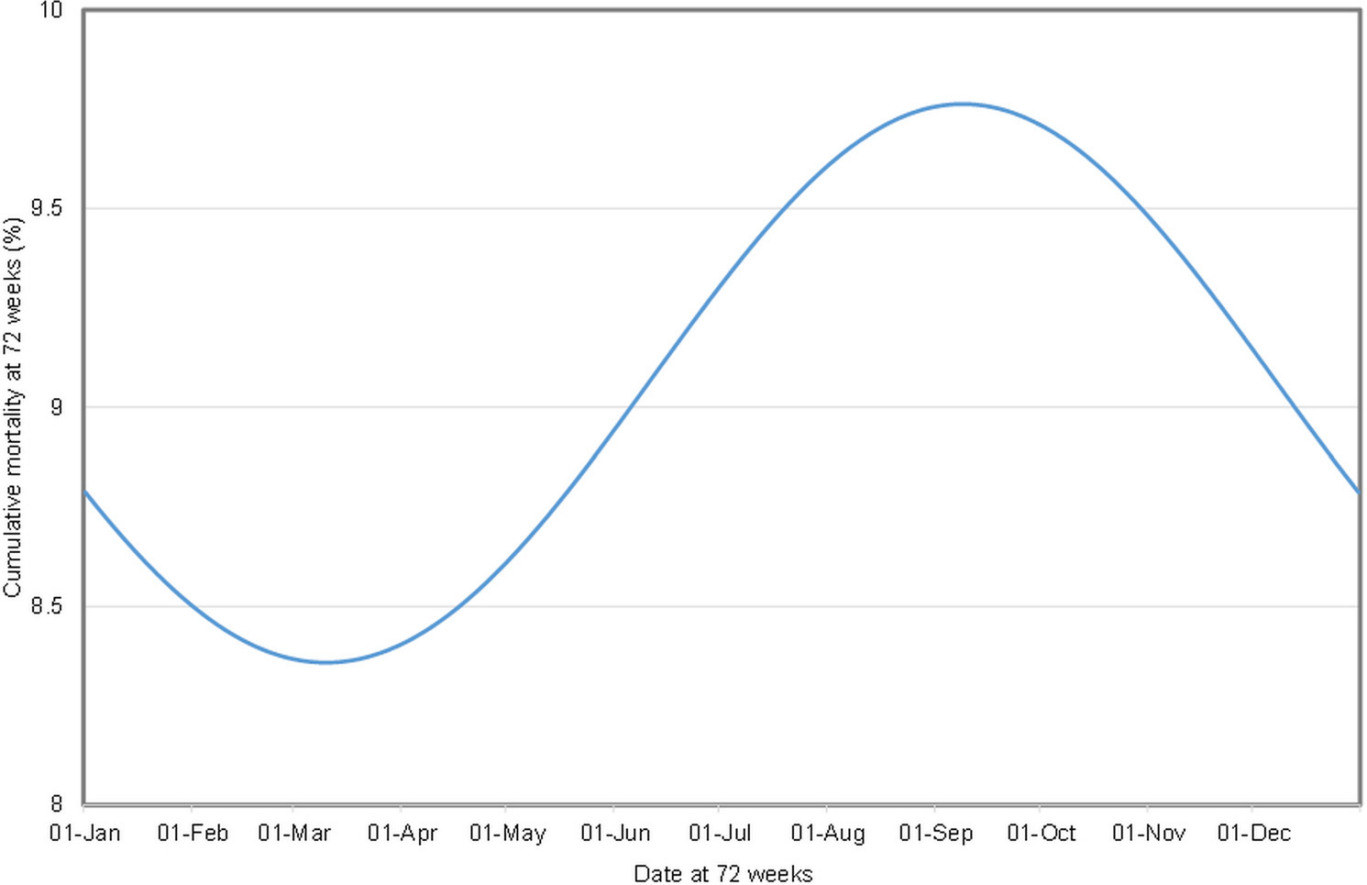
Predicted CM for a free range flock reaching 72 weeks at different times of year (modelled from 2,848 flocks, on 1,649 farms, from six studies).

**Fig 3:**
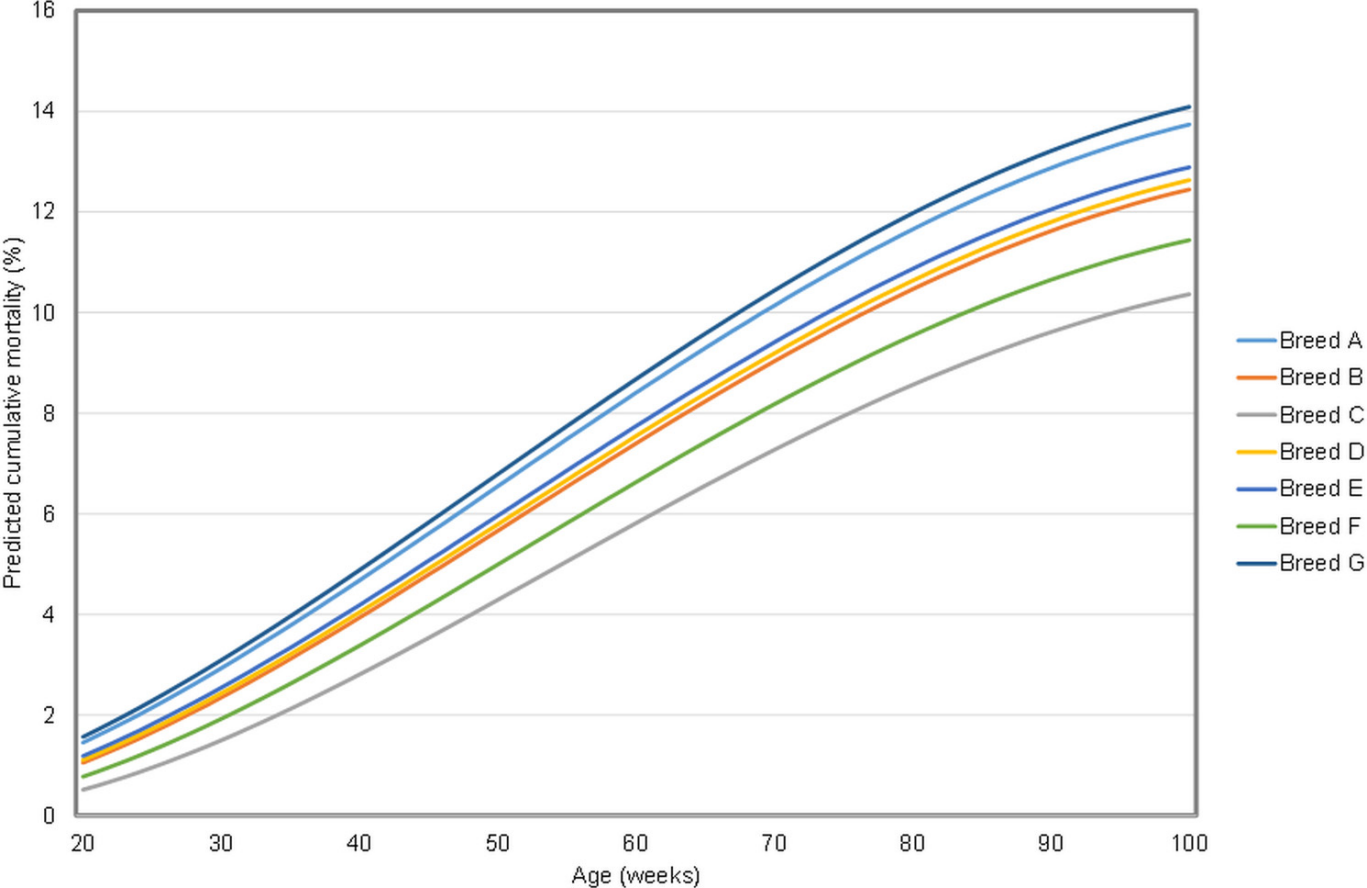
Shows the predicted change in mortality over the lifetime of a flock for each breed (or genotype group) represented in the dataset. Predictions were generated assuming a date of 1st July 2012 for a free range housing system.

In the final model, which explained 84.9% of the variation in the dataset, and included 3,132 data points (from 2,848 flocks, on 1,649 farms, from six studies), CM increased with age in a quadratic relationship (χ^2^ = 459.9; df 2; p<0.001) and varied with: time of year (Fig 2; χ^2^ = 23.5; df 2; p<0.001); breed p<0.001; (Fig 3; χ^2^ = 103.2; df 6); and housing system (χ^2^ = 62.9; df 4; p<0.001;). This model excluded data from studies three, four, six and eight, due to missing data fields in those studies. Thus this principal model included only UK data.

The model produced predictions for cumulative mortality, which was highest in free range flocks and lowest in flocks housed in conventional cages. Predicted mean CM for a free range flock at 72 weeks of age varied seasonally and was lowest for a flock reaching that age in early March at 8.4%, and highest for a flock reaching 72 w in early September, at 9.8%. Using predictions based on the predominant breed in the dataset, for 1^st^ July, predicted mean CM in a free range flock was 3.9%, 7.4% and 9.3% at 40, 60 and 72 weeks of age. In comparison, for a flock housed in a conventional cage system, the corresponding mean CM were 1.7%, 4.2% and 5.7%, respectively.

There was some evidence for an association between CM and beak trim status; CM was higher in flocks with intact beaks (χ^2^ = 3.84; df 1; p = 0.050), when beak trim status was included in the final model, described above. At 40 weeks of age the most common intact beak breed in this dataset, kept in a free range system had a predicted mean CM in intact beak flocks of 3.20% vs. 2.52% in beak trimmed flocks: the figures for 70 w of age are predicted at 8.30% and 7.17% respectively. This model explained 91.6% of the variation in the dataset; however, information on beak trim status was only available for 851 of the 3,132 observations in the principal model. Consequently the principal model (above) is presented without beak trim status. Nonetheless, breed remained a significant predictor in a model with beak trim status (χ^2^ = 18.2; df 6; p = 0.006), while housing system retained a slight association (χ^2^ = 4.04; df 2; p = 0.133). Beak trim information was only available for barn, free range and free range aviary systems, and these two variables were heavily confounded, with 204 of the 206 observations from intact beaked flocks also coming from free range systems (including both organic and non-organic).

There was limited evidence for an effect of organic status. When the housing system variable was expanded to include free range organic and free range aviary organic (that is seven categories, as opposed to the five explored by the housing system variable in the principal model) pairwise comparisons did not suggest that there was a significant difference in CM between free range and free range organic flocks. As there was only one free range organic aviary flock a robust comparison was not possible for this category.

One sensitivity analysis was conducted by running the principal final model again with all flocks of <500 birds excluded from the model. This model contained 3,070 data points, and the relationships between the variables remained the same. A second sensitivity analysis was conducted by running the final model again with all flocks of >100 weeks of age excluded from the model. This model contained 3,118 data points, and again, the relationships between the variables were unchanged. Finally, in a model based on 3,115 data points, which excluded all data points where CM was >40%, all relationships remained unchanged. When all of the above conditions were applied at once, 3,051 data points remained, and all relationships were unchanged.

### 3.2. Environmental impacts

**Fig 4:**
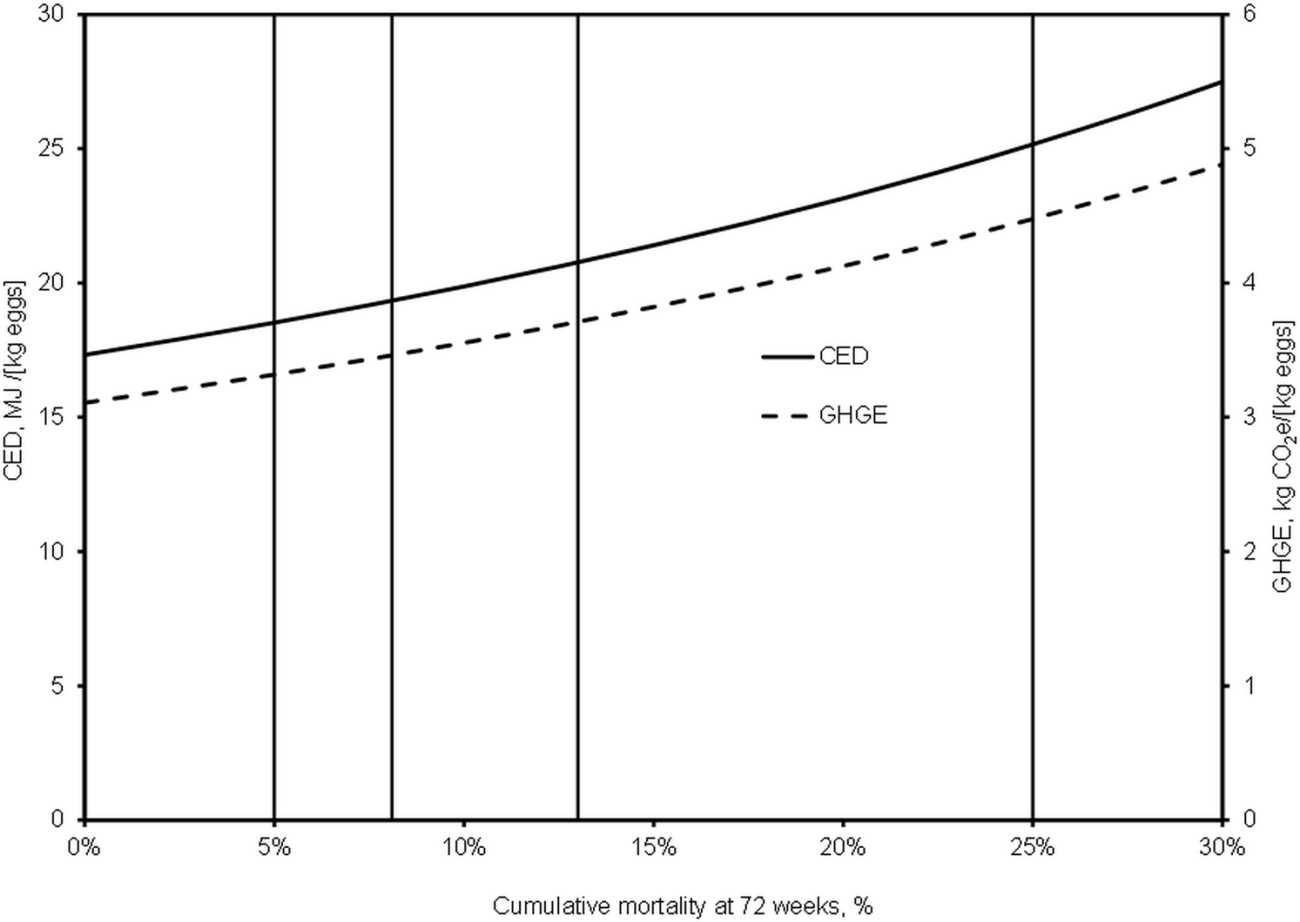
Effects of increasing levels of cumulative mortality at 72 weeks on cumulative energy demand (CED) and greenhouse gas emissions (GHGE) for free range egg production. Levels of up to 30% are modelled. The vertical gridlines show values for the 1st quartile, median, 3rd quartile and upper adjacent value associated with the mean CM of 10%.

The general effect of increasing levels of mortality at 72 weeks is to increase all environmental impacts, with the examples of cumulative energy demand (CED) and greenhouse gas emissions (GHGE) shown in Fig 4. Impacts increase non-linearly, with an ever increasing slope, from a hypothetical base of zero mortality by about 7% at the 1^st^ quartile (5%), 12% at the median (8.1%), 15% at 10% CM (the mean value for free range hens between 60–80 weeks of age), 20% at the 3^rd^ quartile (13%) and 45% at 25% CM (upper adjacent value).

Similar relationships applied to all other impact categories, i.e. acidification potential, eutrophication potential, abiotic resource use, pesticide use and land occupation, which could be described by quadratic relationships, although these were close to linear up to 20% CM (Table 3).

**Table 3:** Relationships between cumulative mortality (CM) and environmental burdens for free range egg production. The functional unit is 1 kg collected eggs.

CM, %	CED, MJ	GHGE kg CO_2_e	Eutroph-ication potential, g PO_4_^3-^ Eqv.	Acidification potential, g SO_2_ Eqv.	Pesticide use, dose-ha * 10^−3^	Abiotic resource use, g Sb Eqv.	Land occup-ation, m^2^.yr
0	17.3	3.11	54.9	20.3	2.05	13.5	4.44
5	18.5	3.32	58.2	21.5	2.17	14.5	4.71
10	19.8	3.56	61.9	22.9	2.31	15.6	5.03
15	21.4	3.83	66.2	24.4	2.47	16.9	5.39
20	23.1	4.13	70.9	26.2	2.64	18.4	5.78
25	25.0	4.46	76.1	28.1	2.83	20.0	6.22

With average CM of 10%, the proportion of CED contributing to pullet production is 17%. As CM increases, the flock produces fewer eggs and hence the proportion of resources going into pullet rearing increases (up to 20% at the upper adjacent value of CM). More pullets are needed *per se* as well as relatively, and the feed needed by hens to produce the same quantity of eggs also goes up. Further, inputs of physical resources, such as electricity, increase per unit egg production. The total feed weight in, per unit egg weight out (the whole system feed conversion ratio) increases from 2.9 at a theoretical zero mortality to 4.1 at 25% CM (Table 4) and electricity consumption goes up from 0.19 to 0.31 kWh per kg eggs.

**Table 4:** Effect of cumulative mortality (CM) on whole system feed conversion efficiency (including breeding overheads and the production phase) and on typical electricity use.

CM, %	Whole system feed conversion ratio, (kg feed/kg eggs)	Electricity, kWh/kg eggs	Meaning of the CM value used
0	2.9	0.19	
5	3.1	0.21	1^st^ Quartile
8	3.2	0.21	Median
10	3.3	0.22	Mean
13	3.4	0.23	3^rd^ Quartile
25	4.1	0.31	Upper Adjacent Value
60	7.8	0.70	Maximum value in data

#### 3.2.1. National scale implications

Using the CM values predicted by the models discussed above, the effects of changes in CM in free range flocks on environmental burdens were investigated. The greenhouse gas emissions (GHGE) of the UK’s free range flock (44% of production) are about 930 kt CO_2_e per annum with current average mortality of 10%. The additional environmental cost of these current average levels of mortality in free range systems is therefore 100 kt CO_2_e per annum, i.e. if zero mortality was possible up to 72 weeks. This rises by a further 35 kt CO_2_e at 13% CM (3^rd^ quartile) and at 25% CM (upper adjacent value) becomes 330 kt CO_2_e above zero CM. Such high mortalities are fortunately not commonplace, but our data show that they do occur (Fig 1) and the analyses presented indicate the potential scale of the effect of very poor management, housing or bio-security. The potential for reducing GHGE in the current average flock is a maximum reduction of 12%, but the target of zero mortality is unrealistic (Table 5). A potentially more achievable reduction in CM from the current mean to the current 1^st^ quartile would reduce total emissions on those farms by 6%. Greater impact might, however, be derived from the poorer performers reducing their mortalities to the current average. Reducing CM from the 3^rd^ quartile to the 1^st^ quartile would reduce GHGE by 9%. In the extreme case of farms operating at the upper adjacent value (25% CM), reducing mortalities to the lower quartile would reduce GHGE by 25%.

**Table 5:** Baseline environmental burdens (i.e. for FR hens up to 72 weeks with CM at 10%) and the change (absolute and percentage) in these burdens if all mortalities in breeder hens, pullets and laying hens are eliminated.

	CED, MJ kg^-1^	GHGE, kg CO_2_e kg^-1^	Eutrophication potential, g PO_4_^3-^ Eqv. kg^-1^	Acidification potential, g SO_2_ Eqv. kg^-1^	Pest-icides used, Milli-dose-ha kg^-1^	Abiotic resource use, g Sb Eqv. kg^-1^	Land occupation, m^2^ yr^-1^ kg^-1^
Baseline value	17.3	3.11	20	55	2.1	13	4.45
Value if all mortalities are eliminated	19.8	3.53	23	62	2.3	15	5.01
% change in burden if all mortalities are eliminated	12%	12%	11%	11%	11%	13%	11%

#### 3.2.2. Sensitivity analysis

In all cases except egg yield, the responses of environmental burdens to 10% increases in feed intake, hen mortality, on-farm energy, pullet mortality and breeder mortality were linear, i.e. with the positive and negative changes being of equal magnitude. Egg yield was close to linearity with the response to an increase in yield of 10% having 94% of the magnitude of a decrease by 10%. The model is most sensitive to egg yield, followed by feed intake (Table 6) and indicates that egg yield has a uniform effect across all burdens. The effects of hen mortality are an order of magnitude lower than egg yield, with on-farm energy similar for cumulative energy demand and greenhouse gas emissions, but much lower for other impacts. Egg yield is correlated with both mortality and feed intake. The impact of pullet and breeder hen mortality was another order of magnitude lower than hen mortality.

**Table 6:** Sensitivity analysis of the LCA model showing response (% change) in environmental burdens to an increase in input variables by 10%.

Variable increased by 10%	Response to 10% increase						
	CED	GHGE	EP	AP	Pest	ARU	Land
Egg yield	-7.8%	-7.8%	-7.8%	-7.8%	-7.8%	-7.8%	-7.8%
Feed intake	5.2%	6.0%	8.3%	8.1%	8.4%	3.3%	7.3%
Hen mortality (i.e. to 11%)	1.3%	1.3%	1.2%	1.2%	1.2%	1.4%	1.2%
On-farm energy	1.1%	0.38%	0.00%	0.10%	0.00%	0.58%	0.00%
Pullet mortality	0.01%	0.01%	0.00%	0.01%	0.01%	0.01%	0.01 %
Breeder mortality	0.01%	0.01%	0.01%	0.01%	0.01%	0.01%	0.01%

## Discussion

The final model of CM based on 3,132 data points from 2,848 flocks, on 1,649 farms, showed that the rate of mortality increased with age. This is consistent with the performance standards in breeder manuals, which all show increases in the rate of CM with time. The predicted patterns of mortality shown in Fig 3 suggests that a more rapid increase in CM over time occurs after about 30–35 weeks of age. The fitted model had a quadratic shape, with a slight tendency to flatten when approaching 100 weeks. This represents whole flock mortality rather than the liveability of an individual bird. It is highly likely that the flocks which are kept up to 100 weeks are those in which mortalities are successfully minimised such that they are economically viable. Flocks with very high CM are more likely to be depopulated after a shorter period, e.g. at about 60 weeks. It is also notable that our data are much sparser between 80 and 100 weeks of age; consequently predictions at this extreme should be interpreted with caution.

Consistent with other surveys and reviews [3–7, 11, 24], we also found that CM differed between housing system, with free range systems predicted to have the highest mortality and conventional cage systems the lowest. There was evidence that CM differed between breeds of hen as was also found by [25]. Sensitivity analyses suggest that the results of this model are robust, since they are not affected by the removal of outliers. Performance standards in breeder manuals similarly show inter-breed variation, with a tendency towards lower mortalities in caged than in free range systems.

Where studies have not found statistically significant differences in the levels of mortality between housing systems this may be due to insufficient power of the dataset. For example a recent comparison of 9 cage flocks with 8 flocks housed in barns in Canada found a consistent trend at all ages for lower mortality in cages than barns but this did not reach statistical significance [26]. A systematic review [25] relied on comparing mean levels of CM in conventional cages with those in aviaries for each study, and further averaged this variable to an unweighted mean over 4 weeks that might not therefore have reflected flock variability and the rate of increase of CM with age.

Interestingly, CM showed a significant association with time of year. When CM in 72 week old flocks was modelled for various times of year it was lowest in early March and highest in early September. A 72 week old flock will have been housed for a little over a year, assuming flocks are transferred from rear to lay at around 16 weeks of age. Thus a flock which reaches 72 weeks in early March will have been housed in early February of the preceding year, and a flock reaching 72 weeks of age in early September, will have been housed in early August the preceding year. We did not find any interaction between time of year and housing system, thus this seasonal effect appears to be uniform across housing systems. It may be that, regardless of housing system, flocks which arrive in their laying house in early spring experience warmer temperatures during their peak laying period, meaning that the birds are under less physiological stress, since they require less energy to keep warm.

There was some evidence that CM was reduced in beak trimmed flocks kept in free range housing systems (the only housing system for which a number of beak trimmed and intact beak flocks were represented). Predicted means for breed B suggest that, for example, levels of CM may be 27% higher in an intact beak flock at 40 weeks of age. Beak trimming is used as a means to reduce the damage inflicted from injurious pecking, which has been associated with increased mortality [27–29], so this difference is not unexpected. There was no robust evidence for a difference in CM between organic and non-organic free-range flocks.

Importantly, although the mean CM for free range flocks is higher, there was also more variability in CM for this housing system; in the 579 free range flocks for which we had data at 72 weeks of age the lower quartile of CM ranged from 0.6% to 5.0% while the upper quartile ranged from 11.6% to 53.3% (Fig 1). This suggests that with good management there is considerable scope for most free range flocks to achieve lower levels of mortality, more comparable to those found in cage systems. There is a need for more research to understand the effects of housing system, genetics and management on mortality. Specifically, it would be of interest to determine the causes of mortality in different housing systems and which differences between genotypes are associated with the various risks for mortality. It is possible, for example, that some breeds are more susceptible to disease or climate stress, whereas others are more susceptible to injurious pecking. Furthermore it is important that we understand the interaction between genetics and the environment, with the possibility that breeds can be developed for different systems and also be better managed within systems. Whilst flocks housed in cage systems generally achieve low mortalities similar to the best loose-housed flocks, there are higher costs associated with equipment (steel cages) and fan ventilation that may offset this, together with the compromises of reduced behavioural repertoire and bird choice of environment. In characterising and comparing housing systems for laying hens, the EU Laywel project [2] recognised that no one system was ideal in every aspect; without considering the environmental impacts that have emerged in recent years as of importance. Indeed, a recent review highlighted the need for further research to evaluate the environmental impacts of all housing systems, while at the same time recognising the effects within systems of design, operation and management on actual emissions and the ‘environmental footprint’ [30].

This study has not addressed the economic effects of varying mortality but a comparative economic assessment of three housing systems [31] found that the higher costs of egg production in a loose-housed (aviary) system compared with conventional cage and enriched cage systems were in part due to increased levels of mortality and reduced productivity; moreover it was more expensive to rear pullets in the enriched barns appropriate for aviary or free-range production than in cages. The environmental effects we were able to predict from the data available are most probably an underestimate. Flocks with high mortality are likely to have underlying high morbidity and hence yield depression in both conspecifics and the casualties before they die. This alone would increase the inputs of resources such as feed (particularly in birds with feather loss) during periods of reduced egg production, as birds will still need feed to support their basal metabolic needs and possibly fight infections. Hence, the balance of resources used (and emissions generated) would further increase environmental impacts per unit output. Our estimations of high levels of mortality on sustainability are model-based. The LCA model was developed using industry average data, which does not necessarily reflect all aspects of the performance of high CM flocks. There is scope for a more rigorous evaluation by recording the activity data (e.g. feed and energy use and productivity) on farms with high CM to validate the assumptions in the systems-LCA model. A combined study to determine causes of CM and measuring the effects of improved management would be valuable. Such a study should also include economic analysis to address sustainability more fully.

High levels of mortality are unsustainable and unethical. It has been argued [32] that poor liveability of a flock not only suggests bird health problems but also a poor welfare state in morbid birds. The principal causes in free-range laying hens are disease, predation, injurious pecking and smothering [24, 32]; they are affected by many risk factors, not all of which are well understood. They are all complex issues that are difficult to manage, and reducing them requires a multi-faceted approach. There is potentially scope to further select bird genotype in order to reduce levels of mortality in loose-housed systems. As shown in Fig 3, our model predicts up to a two-fold difference between genotypes in flock mortality at 100 weeks of age; this is the lifespan for which breeding companies are currently selecting, due to economic pressure to maintain birds in productive lay for longer [33]. Among several other possible approaches to reducing the levels of mortality are refinements in housing design (e.g. [24]).

The level of mortality in a flock is, therefore, a useful quantitative indicator not only of bird welfare (and thereby of consumer expectation) but also impacts other aspects of sustainability such as the environmental footprint and economic viability of the production system. Hence, as we have indicated in this paper, reducing levels of premature mortality is likely to have multiple benefits.

## Conclusions

High levels of mortality reduce the sustainability of egg production (for example reducing the levels in free-range systems to those currently achieved by the best quartile could reduce GHGE by up to 25% and save resources such as land, feed and fuel). Concurrently this would enhance hen welfare and better meet the expectation of egg consumers. The indication that there may be genetic risk factors associated with susceptibility to mortality in free range systems in particular, highlights the need for more research to understand the genetic x environment interaction. More detailed records of the causes of mortality are also required so that improved genotypes can be developed for different systems and different breeds can be better managed within systems.
